# Automated Scoring of Narrative Recall Assessments Using Large Language Models Enables Exploration of Alternate Scoring Criteria

**DOI:** 10.1002/alz70857_099418

**Published:** 2025-12-24

**Authors:** Michael J Kleiman, Katana Rader, Simone Camacho, James E Galvin

**Affiliations:** ^1^ University of Miami Miller School of Medicine, Boca Raton, FL, USA; ^2^ University of Miami Miller School of Medicine, Miami, FL, USA

## Abstract

**Background:**

Clinical neuropsychological assessments, particularly narrative recall tests, present significant challenges for real‐time scoring due to the need for rapid interpretation. While post‐hoc scoring from recordings improves accuracy, time constraints in clinical settings often preclude this approach. Large Language Models (LLMs) have demonstrated promise in scoring verbal responses, potentially offering improved reliability and consistency compared to traditional human rating methods.

**Method:**

28 participants (14 MCI, 14 age‐matched CN), completed the Craft Story 21 assessment, immediate and delayed. ChatGPT‐4o was provided human‐corrected transcripts along with two sets of questions; a standard scoring criteria which matched that used by human raters, and a novel set of questions, both utilizing few‐shot and chain‐of‐thought prompting strategies.

**Result:**

LLM scoring demonstrated moderately high correlations with human raters for both immediate (*r* = 0.654, *p* < .001) and delayed recall (*r* = 0.655, *p* < .001). Interrater reliability was comparable between LLM (ICC2k=0.580, *p* = .015) and human raters (ICC2k=0.574, *p* = .008). LLM scoring showed superior discrimination of cognitive status for immediate recall (t(26)=2.64, AUC=0.765, *p* = .013) compared to human scored responses which did not find any difference (t(26)=1.26, AUC=0.638, *p* = .218), while discrimination in human‐scored assessments was significantly better for the delayed recall responses (t(26)=2.50, AUC=0.747, *p* = .019) than LLMs (t(26)=1.57, AUC=0.662, *p* = .128). A novel set of questions was then developed to separate general from specific answers, with variable weighting per question, which exhibited an ability to improve discrimination in the immediate recall assessment (AUC=0.798, *p* = 0.010) compared to the standard questions, but not in the delayed component.

**Conclusion:**

LLMs demonstrate potential as a reliable, cost‐effective alternative to human scoring, offering consistent performance and the ability to retrospectively apply modified scoring criteria for research purposes. However, the presence of hallucinations and score variability between model runs suggests that this technology is at a premature stage for use in clinical settings. Nonetheless, rapid advancements in the field may result this strategy being a viable alternative in the very near future, especially as more advanced reasoning models become more available and consistent.

